# Effects of physical activity on the link between PGC-1a and FNDC5 in muscle, circulating Ιrisin and UCP1 of white adipocytes in humans: A systematic review

**DOI:** 10.12688/f1000research.11107.2

**Published:** 2017-05-26

**Authors:** Petros C. Dinas, Ian M. Lahart, James A. Timmons, Per-Arne Svensson, Yiannis Koutedakis, Andreas D. Flouris, George S. Metsios

**Affiliations:** 1 Institute of Sport, Faculty of Education Health and Wellbeing, University of Wolverhampton, Walsall, WS1 3BD, UK; 2 FAME Laboratory, Department of Physical Education and Exercise Science, University of Thessaly, Trikala, GR42100, Greece; 3 Genetics and Molecular Medicine, King’s College London, London, SE1 9RT, UK; 4 Department of Molecular and Clinical Medicine, The Sahlgrenska Academy, University of Gothenburg, Gothenburg, SE-413 45, Sweden; 5 Department of Physical Education and Exercise Science, University of Thessaly, Trikala, GR42100, Greece; 6 Institute for Research and Technology, Trikala, GR42100, Greece

**Keywords:** Exercise, FNDC5, Irisin, UCP1

## Abstract

**Background: **Exercise may activate a brown adipose-like phenotype in white adipose tissue. The aim of this systematic review was to identify the effects of physical activity on the link between peroxisome proliferator-activated receptor gamma coactivator 1-alpha (PGC-1a) and fibronectin type III domain-containing protein 5 (FNDC5) in muscle, circulating Irisin and uncoupling protein one (UCP1) of white adipocytes in humans. **Methods: **Two databases (PubMed 1966 to 08/2016 and EMBASE 1974 to 08/2016) were searched using an appropriate algorithm. We included articles that examined physical activity and/or exercise in humans that met the following criteria: a) PGC-1a in conjunction with FNDC5 measurements, and b) FNDC5 and/or circulating Irisin and/or UCP1 levels in white adipocytes. **Results: **We included 51 studies (12 randomised controlled trials) with 2474 participants. Out of the 51 studies, 16 examined PGC-1a and FNDC5 in response to exercise, and only four found increases in both PGC-1a and FNDC5 mRNA and one showed increased FNDC5 mRNA. In total, 22 out of 45 studies that examined circulating Irisin in response to exercise showed increased concentrations when ELISA techniques were used; two studies also revealed increased Irisin levels measured via mass spectrometry. Three studies showed a positive association of circulating Irisin with physical activity levels. One study found no exercise effects on UCP1 mRNA in white adipocytes. **Conclusions:** The effects of physical activity on the link between PGC-1a, FNDC5 mRNA in muscle and UCP1 in white human adipocytes has attracted little scientific attention. Current methods for Irisin identification lack precision and, therefore, the existing evidence does not allow for conclusions to be made regarding Irisin responses to physical activity. We found a contrast between standardised review methods and accuracy of the measurements used. This should be considered in future systematic reviews.

## Introduction

Brown adipose-like phenotype in white adipose tissue (WAT) may play a role in reducing body weight, and consequently lessen obesity in mammals ^1^. Recently, acute and chronic exercise has been found to induce a brown adipose-like phenotype in WAT ^2^ through a number of sequential steps. Exercise is also known to increase the activation of the peroxisome proliferator-activated receptor gamma coactivator 1-alpha (PGC-1α) gene in human skeletal muscle ^3^. PGC-1α is a co-transcriptional regulator facilitating multiple transcription factors to regulate a complex network of genes ^4^ and it has been implicated in both the control of tissue mitochondrial content and the program that results in brown adipose tissue (BAT) formation ^5^.

While skeletal muscle properly adapts to exercise in the absence of PGC-1α ^6^, activation of PGC-1α was proposed to increase the fibronectin type III domain-containing protein 5 (FNDC5) ^2^. FNDC5, is a membrane protein expressed in brain and skeletal muscle ^7^. It was proposed that FNDC5 was cleaved during exercise, and released into the bloodstream as Irisin – a peptide fragment of FNDC5 measured by western blotting ^2^. *In vitro*, exposure of white adipocytes to Irisin– through an unknown receptor – subsequently led to an increase of the peroxisome proliferator-activated receptor alpha, which in turn increased uncoupling protein one (UCP1) mRNA ^2^. The increase in white adipocyte UCP1 mRNA observed with Irisin treatment, presented as fold-change over control, is hard to interpret since white adipocytes in culture do not usually express UCP1 mRNA ^8^.

Since, UCP1 is the only contributor to non-shivering thermogenesis that occurs in BAT ^9^ and it appears that the presence of UCP1 in a white adipocyte is accompanied by “brown-adipocyte like” properties ^8, 10, 11^, it was proposed that increased circulating Irisin in humans after a chronic exercise program may promote increased weight loss and improved metabolic control through induction of UCP1 ^2^. This hypothesis seemed superficially plausible, as Irisin over-expression stimulated oxygen consumption and has been described to have an inverse association with blood glucose, insulin, total cholesterol and a positive association with adiponectin concentrations ^12^. However, other studies have failed to observe such positive associations ^13– 15^, while the effect of exercise on “browning” of the white adipose phenotype remains unclear ^16– 18^.

The exact role of exercise in regulating circulating Irisin concentration remains to be established. Indeed, data indicate that while older adults appear to have a 30% increase in FNDC5 mRNA in muscle compared to younger adults, FNDC5 mRNA was unresponsive to six weeks of endurance training ^19^, despite robust increases in mitochondria ^20^. In general, results on the effects of exercise on circulating Irisin ^17, 21– 24^ have been rather ambiguous; diverse methodology may explain the highly discrepant results ^25, 26^. Given that Irisin continues to be measured using a variety of methods, an evaluation of the available evidence for its relationship with humans’ health is warranted, due to the potential that the browning of white adipocytes may have on human health. In addition, the proposed exercise mechanism that may cause a browning process of WAT in humans must be evaluated. Therefore, the aim of the current review was to systematically identify the effects of physical activity on the link between PGC-1α and FNDC5 in muscle, and circulating Irisin, as well as evidence for regulation of UCP1 in WAT (indicating a browning process) in humans.

## Methods

### Search strategy

Using the Preferred Reporting Items for Systematic Reviews and Meta-Analyses (PRISMA) guidelines ^27– 29^, two databases (PubMed and EMBASE) were searched up until 19 ^th^ August 2016. Two investigators (PCD and IML) independently conducted two identical searches in both databases using appropriate search algorithms (PubMed: Supplementary File 1; EMBASE: Supplementary File 2). The lists of the included articles were reviewed to identify publications that were relevant to the topic under review.

### Selection criteria

We included studies that met at least one of the following eligibility criteria: a) measurements of PGC-1a (mRNA and/or protein concentrations) in conjunction with measurements of FNDC5; b) measurements of FNDC5, and/or Irisin concentrations and/or UCP1 in WAT, along with the following criteria: c) measurements of physical activity levels and/or exercise interventions, and d) human participant study. No other eligibility criteria were set (e.g., language, date of publication). From the included studies, we retrieved outcomes regarding the effects of physical activity on PGC-1a in conjunction with FNDC5 in muscle, FNDC5 in muscle, Irisin in the bloodstream and UCP1 in WAT. We report the studies’ design, the participants’ characteristics, the Irisin identification and other outcome methods and study outcomes. We have also recorded the secondary associations in the included studies, i.e. associations between FNDC5 and/or circulating Irisin and several health-related phenotypes [e.g. energy expenditure, blood pressure, waist to hip ratio, body mass index (BMI)].

### Risk of bias assessment and quality of reporting data

Two independent reviewers (PCD and GSM) evaluated the risk of bias of the studies included in the current review via the “Cochrane Collaboration’s tool for assessing risk of bias” ^30^. Conflicts in the risk of bias assessment were resolved by IL and ADF. We also evaluated independently (PCD and GSM) the quality of reporting in the included randomised controlled trials (RCTs), controlled trials (CTs) and single group design studies (SGS) using the Consolidated Standards of Reporting Trials (CONSORT) checklist ^31^, which is a 25-item checklist and we provided a score for each study included. For CTs and SGS, we used a modified CONSORT checklist comprised of 18 items, given that these studies are not RCTs and therefore, seven out of the 25 items of the CONSORT checklist are not applicable for CTs and SGS (i.e. randomization, blinding). We also evaluated independently (PCD and GSM) the quality of the reporting data of the included cross sectional studies (CSS) using the 22-item checklist of the Strengthening the Reporting of Observational Studies in Epidemiology (STROBE) and we also provided a score for each study included ^32^. Disagreements on studies’ CONSORT and STROBE scores were arbitrated by IL and ADF. JT and PS then reviewed the molecular and genomic content of the review independent of the search process.

## Results

The reporting of the available information in this systematic review is shown in a PRISMA checklist in Supplementary Table 1.

### Searching procedure results

The initial searching date was the 14 ^th^ September 2015 while weekly alerts were received from both databases up until the 19 ^th^ August 2016. Overall, the searching procedure revealed 51 studies that involved 2474 participants and met the inclusion criteria, and were therefore included in this systematic review. The reference lists of these studies did not result in the identification of additional relevant articles. The searching outcome is presented in a PRISMA flow diagram in Supplementary Figure 1.

### Characteristics of the included studies

The characteristics and the results of the included studies can be found in Table 1. From the 51 eligible studies, 12 (23.5%) were RCTs, of which four were cross-over RCTs, eight (15.7%) were CTs, 23 (45%) were SGS, and eight (15.7%) were CSS. One of the included RCTs ^33^ reported the effect of resistance exercise training versus the effects of resistance exercise training combined with Ursolic supplementation, because for the latter group the effects of resistance exercise cannot be isolated, we will report only the results from the resistance exercise training group. Furthermore, one of the CTs ^34^ will be included in the results of both CTs and CSS because this study consisted of a controlled trial nested within a CSS. Eight of the included studies examined overweight/obese adults and children ^17, 35– 41^, while 11 studies included a clinical population, including patients with chronic obstructive pulmonary disease (COPD) ^23, 34, 42^, heart failure ^43^, metabolic syndrome ^44^, haemodialysis ^45^, osteoporotic ^46^, anorexia nervosa ^36, 47^, pre-diabetes ^16^ and diabetes type II ^48^.

**Table 1.** Characteristics of the studies included in the systematic review.

First author	Design	Participants	Main outcome	Secondary outcome	Method of circulating Irisin identification
**PGG-1a and FNDC5**					
***Acute exercise***					
Nygaard, 2015	C-RCT	Two F and seven M moderately trained healthy	Aerobic exercise (2.1±0.8-fold over baseline,p=0.05) and resistance exercise (3.5±0.9- fold over baseline, p=0.01) increased PGC-1a splice 1 mRNA in muscle. No changes on FNDC5 mRNA in muscle. No correlations between PGC-1a splice variant 1 mRNA in muscle and Irisin.	NA	NA
Norheim, 2014	CT	13 M healthy controls, and 11 M pre-diabetic	AE increased PGC-1a mRNA in muscle in both groups (7.4-fold over baseline).	NA	NA
Pekkala, 2013	CT	Healthy M: 17 middle-age, 10 young, 29 older	AE increased PGC-1a mRNA in muscle (4- fold in young/2-fold in older over baseline). AE increased FNDC5 mRNA in muscle (1.4-fold over baseline, 95% CI=0.3-2.2) in young.	NA	NA
Camera 2015	SGS	Eight healthy trained M	AE increased PGC-1a mRNA in muscle 4-hour post exercise (200%, p<0.05 over baseline and over control p<0.05), but it did not alter FNDC5 mRNA in muscle.	NA	NA
Kurdiova, 2014	SGS	Sedentary overweight/obese: 10 M, Six F	AE increased PGC-1α mRNA in muscle (>6-fold over baseline), but it did not alter FNDC5 mRNA in muscle.	NA	NA
Lecker, 2012	CSS	24 M systolic heart failure patients	PGC-1a mRNA was positively correlated with FNDC5 mRNA in muscle (r=0.56, p<0.05).	NA	NA
***Chronic exercise***					
Norheim, 2014	CT	13 M healthy controls, and 11 M pre-diabetic	CE increased PGC-1a mRNA (1.2-fold in controls/1.6-fold in pre-diabetic over baseline) and FNDC5 mRNA (1.4-fold in controls/2-fold in pre-diabetic over baseline) in muscle. PGC-1a and FNDC5 mRNA in muscle was positively correlated (r=0.82, p<0.01) when data of both groups were combined.	NA	NA
Pekkala, 2013	CT	Healthy M: 17 middle-age, 10 young, 29 older	21 weeks of CE did not alter PGC-1a, FNDC5 mRNA in muscle.	NA	NA
Timmons, 2012	CT	24 young sedentary and 43 healthy M	6 weeks of CE (intense cycling and resistance) did not alter FNDC5 mRNA in muscle.	FNDC5 mRNA in muscle was not linked to diabetes status.	NA
Alvehus, 2014	SGS	17 healthy young M	8 weeks of CE did not alter PGC-1a mRNA in both muscle and WAT and FNDC5 mRNA in muscle.	NA	NA
Besse-Patin, 2014	SGS	11 sedentary obese M	8 weeks of CE did not alter FNDC5 mRNA in muscle.	NA	NA
Boström, 2012	SGS	Eight non-diabetic M	10 weeks of CE increased FNDC5 mRNA in muscle (p<0.05)	NA	NA
Ellefsen, 2014	SGS	18 untrained young F	12 weeks of CE decreased PGC-1a Slice4 mRNA in muscle (p<0.05), but it did not alter FNDC5 mRNA in muscle.	NA	NA
Huh, 2014	SGS	Healthy: 78 M, 15 M and 15 F adolescents	8 weeks of CE increased PGC-1a mRNA in muscle and FNDC5 mRNA in muscle (p<0.05).	NA	NA
Kurdiova, 2014	SGS	Sedentary overweight/obese: 10 M, Six F	12 weeks of CE did not alter FNDC5 mRNA in muscle	NA	NA
Raschke, 2013	SGS	13 healthy M	A 10-week and 11-week program of CE did not alter FNDC5 mRNA in muscle.	The FNDC5 gene displays a non-ATG start codon and it was not activated by electrical stimulation.	NA
Scalzo, 2014	SGS	Healthy: Seven M, 12 F	3 weeks of CE did not alter FNDC5 mRNA in muscle	NA	NA
**IRISIN**					
***Acute exercise***					
Nygaard, 2015	C-RCT	Two F and seven M moderately trained healthy	Aerobic exercise (p=0.037) and resistance AE (p<0.001) increased Irisin. No correlations between Irisin and PGC-1a splice variant 1 mRNA in muscle.	NA	PP, EK-067-29
Huh, 2015	C-RCT	Eight healthy sedentary M, 4 M with MetS	AE (high density aerobic and resistance) increased Irisin in healthy and metabolic syndrome patients (p<0.05). Resistance exercise was more effective in increasing Irisin than endurance exercise.	NA	PP, EK-067-52, and EK-067-29
Tsuchiya, 2014	C-RCT	Six young healthy sedentary M	Low-intensity running increased Irisin (p<0.05) immediately after exercise compared with pre-exercise values.	NA	PP, EK-067-52
Tsuchiya, 2015	C-RCT	10 healthy M	Resistance AE increased Irisin (p<0.05) while endurance and combined (endurance + resistance) AE did not alter Irisin.	Irisin was positively correlated with blood glucose (r=0.37, p<0.05), lactate (r=0.45, p<0.05) and plasma glycerol (r=0.45, p<0.05).	PP, EK-067-52
Norheim, 2014	CT	13 M healthy controls, and 11 M pre-diabetic	AE increased Irisin in both groups (1.2-fold over baseline). Irisin was not correlated with PGC-1a, FNDC5 mRNA in muscle and UCP1 mRNA in subcutaneous WAT.	NA	PP, EK-067-52, and EK-067-29
Aydin, 2013	CT	14 obese M, and 14 normal weight M	AE increased saliva Irisin (p<0.05). No changes in serum Irisin.	Serum Irisin was negatively correlated with BMI (r-0.944, p=0.005).	PP, EK-067-52
Kraemer, 2014	CT	Healthy: Seven M, Five F	AE increased Irisin at 54th minute of the exercise session (20.4% compared to baseline, F _(3,36)_=5.28, p = 0.004), but decreased after the exercise session in M (p=0.021). AE increased Irisin at 54 ^th^ minute (F _(3,24)_=5.03, p = 0.008) in F.	NA	AB, Burlingame, CA, USA (CNS)
Huh, 2014	SGS	Healthy: 78 M, 15 M and 15 F adolescents	AE increased Irisin in treadmill (p<0.001) and swimming (p<0.05) conditions.	Irisin was positively correlated with blood lactate (r=0.30, p=0.04). Incubated Irisin in human skeletal muscle cells (in vitro) increased glucose and fatty acid uptake (p<0.05).	PP, EK-067-52
Moienneia, 2016	SGS	21 sedentary young healthy F	AE of both low and high intensity resistance training did not alter Irisin (p>0.05).	NA	ELISA CUSABIO, China
Anastasilakis, 2014	SGS	20 young healthy 10 F and 10 M	AE increased Irisin (p<0.001). No association of PA levels with circulating Irisin.	Irisin was positively correlated with LBM (r=0.28, p=0.02) and glucose (r=0.24, p=0.01) but it was not correlated with BMI, WHR, HOMA, insulin and leptin.	PP, EK-067-52
Comassi, 2015	SGS	14 M ironman racers, 13 M half- ironman races	The half-ironman race increased Irisin (p<0.05).	NA	Not mentioned
Daskalopoulou, 2014	SGS	Healthy: 22 M, 17 F	AE (treadmill) increased Irisin (35% over baseline, p<0.001), with greater increase in maximal workload (p=0.004).	Irisin and lactate were positively correlated with their changes of pre- post exercise after maximal workload (r=0.52, p=0.001). Irisin was positively correlated with post exercise VO _2_max (r=0.39, p=0.02) but, not with post exercise REE.	PP, EK-067-52
Huh, 2012	SGS	15 healthy M	AE increased Irisin (p=0.001)	Irisin was not correlated with ATP levels after exercise	AB, Santa Clara, CA, USA (CNS)
Huh, 2014a	SGS	14 healthy F	AE (vibration) increased Irisin at both baseline (9%, p=0.05) and post CE (18%, p=0.05).	Irisin was positively correlated with cortisol after exercise (r=0.41, p=0.04).	PP, EK-067-52
Khodadadi, 2014	SGS	21 overweight F	High intense interval AE increased Irisin (33%, p=0.039). One session of Pilates exercise did not alter Irisin.	NA	ELISA CUSABIO, China
Löffler 2015	SGS	28 healthy adults. Children 12 years and older, 48 M, 40 F	AE increased Irisin in both adults (p=0.006) and children (p<0.001).	Irisin was positively associated with BMI (r=0.41, p=0.03), WHR (r=0.57, p=0.010, LBM (r=0.60, p=0.002), blood glucose (r=0.39, p=0.04) and triglycerides (r=0.44, p=0.02) as well as negatively with HDL (r=-0.46, p=0.01) in adults.	PP, EK-067-52
Lee, 2014	SGS	Healthy: Six M, Four F	Submaximal AE increased Irisin (3.1-fold over baseline, p<0.05), whereas maximal AE did not alter Irisin following graded stepwise cold exposure.	Irisin increased after cold exposure and changes in Irisin concentrations positively correlated with shivering activity (r=0.91, p<0.001). REE was greater after maximal exercise compare to cold exposure.	Mass spectrometry/ Western blot BCA-kit/PP, Burlingame, CA, USA (CNS)
***Chronic exercise***					
Bang 2014	RCT	Seven healthy Korean M	8 weeks of CE (resistance) did not alter Irisin.	Exercise did not alter blood glucose and insulin levels.	PP, Burlingame, CA, USA (CNS)
Greulich, 2014	RCT	COPD patients: 26 M, 14 F	8 days of a vibration exercise increased Irisin (p=0.01).	NA	AB, INC. (CNS)
Greulich, 2014a	RCT	22 F and 39 M COPD patients	Three months of CE did not alter Irisin in both non-individualized training group and individualized training group.	NA	AB, INC. (CNS)
Hecksteden, 2013	RCT	Healthy sedentary: 38 M, 64 F	No changes in Irisin after 26 weeks of aerobic exercise.	No relationship between changes in Irisin with age, sex and BMI.	AB, Santa Clara, CA, USA (CNS)
Kim 2015	RCT	40 elderly healthy F	12 weeks resistance CE increased Irisin compared to control group (p<0.05).	Irisin was positively correlated with muscle strength (r=0.526, p=0.002).	PP, USA
Kim, 2016	RCT	17 M and 11 F overweight and obese	8 weeks resistance CE increased Irisin compared to control group (p<0.05).	Irisin was positively associated with muscle mass (r=0.43, p=0.02) and negatively with fat mass (r=0.41, p=0.03)	PP, EK-067-16
Scharhag- Rosenberger 2014	RCT	37 exercised and 34 controls healthy M and F	A 6-month resistance training program increased Irisin in control (p<0.01) but not in exercise group.	Resting metabolic rate was increased in exercise group (p<0.01) but was not associated with Irisin.	PP, Burlingame. CNS and Sunrise microplate reader (Tecan, Mannerdorf, Switzerland)
Tsuchiya, 2016	RCT	20 healthy M	A 4-week sprint CE decreased Irisin (p<0.05).	NA	PP, EK-067-52
Pekkala, 2013	CT	Healthy M: 17 middle-age, 10 young, 29 older	21 weeks of CE did not alter Irisin	Irisin and FNDC5 mRNA in muscle were not associated with HOMA, plasma glucose and serum insulin.	PP, Inc., Burlingame, CA, USA (16–127)
Ijiri 2015a	CT	8 M COPD patients	8 weeks of CE increased Irisin (p<0.05). AE did not alter Irisin.	Irisin was not correlated with pulmonary function parameters and 6- min walk distance.	PP, Burlingame. (CNS)
Miyamoto- Mikami 2015	CT	16 M and nine F young/ 12 M and 16 F middle-aged older healthy	An 8-week CE program increased Irisin in middle-aged/older healthy (p<0.05). Exercise did not alter Irisin in young healthy individuals.	Irisin was negatively correlated with visceral adipose tissue after CE (r=0.54, p<0.05). No correlation of Irisin with abdominal subcutaneous adipose tissue area and whole-body fat.	PP, EK-067-16
Prestes, 2015	CT	72 elderly F	16 weeks of CE (resistance) did not increase Irisin.	NA	MyBioSource Inc., San Diego, CA, USA (CNS)
Ellefsen, 2014	SGS	18 untrained young F	12 weeks of CE did not alter Irisin. Irisin was positively correlated with FNDC5 mRNA in muscle (r=0.65, 95% CI=0.12-0.89, p<0.05).	Irisin was not correlated with fat mass after exercise.	PP, EK-067-29
Kurdiova, 2014	SGS	Sedentary overweight/obese: 10 M, Six F	A 12-week CE did not alter Irisin.	Irisin was negatively associated with fasting glycaemia (r=−0.52, p<0.05) but it was not associated with VO _2_max prior and post exercise.	PP, RK-067-16
Scalzo, 2014	SGS	Healthy: Seven M, 12 F	3 weeks of CE decreased Irisin in M (p<0.05) while it increased Irisin in F (p<0.001). Irisin was not correlated with FNDC5 mRNA.	Irisin was not correlated with fasting glucose, insulin and HOMA.	PP, Burlingame, CA, USA (CNS)
Moienneia, 2016	SGS	21 sedentary young healthy F	An 8-week low intensity resistance training program did not alter Irisin. An 8-week high intensity resistance training reduced Irisin (p=0.034).	NA	ELISA CUSABIO, China
Blüher, 2014	SGS	65 obese children 7–18 years old M and F	12 months of PA intervention increased Irisin (12% over baseline, p=0.00003).	Irisin was not correlated with inflammatory markers at baseline.	PP, EK-067-52
Hew-Butler, 2015	SGS	Nine F non-runners	A 10-week of walk/running program did not alter Irisin.	No relationship of Irisin with LBM, VO _2_peak and fat mass after the exercise program.	PP, Burlingame, CA (EK-067-52 and EK-067-29)
Huh, 2012	SGS	15 healthy M	8 weeks of CE did not alter Irisin.	NA	AB, Santa Clara, CA (CNS)
Huh, 2014a	SGS	14 healthy F	6 weeks of CE (vibration) did not change Irisin.	NA	PP, EK-067-52
Löffler 2015	SGS	28 healthy adults. Children 12 years and older, 48 M, 40 F	6 weeks in-house CE did not alter Irisin in children (n=62). Three years of low grade PA intervention in children did not alter Irisin.	NA	PP, EK-067-52
Moraes, 2013	SGS	13 M, and 13 F haemodialysis patients	A 6-month CE program did not alter Irisin.	Irisin was greater in haemodialysis patients than in healthy at baseline (p<0.05). No correlation with physical capacity, anthropometry and creatinine levels.	PP, Burlingame. (CNS)
Murawska- Cialowicz, 2015	SGS	Seven M and five F healthy	A 3-month cross-fit training program increased Irisin only in F.	Irisin was positively correlated with BMI (r=0.48, p=0.02), fat mass % (r=0.56, p=0.014) and VO _2_max (r=0.43, p=0.012) only in M.	ELISA: BioVendor- Laboratorni Medicina, Czech Republic
Palacios- González, 2015	SGS	85 healthy children 8–11 years old. 45 F and 40 M	An 8-month PA program did not alter Irisin levels.	Irisin was positively associated with BMI before (r=0.78, p<0.001) and after (r=0.82, p<0.001) the PA program as well as leptin (r=0.72, p<0.001) after the PA program.	Cusabio Biotech. (CNS)
Boström, 2012	SGS	Eight non-diabetic M	10 weeks of CE increased Irisin (2-fold over baseline, p<0.05).	Irisin did not alter oxygen consumption and weight loss in vivo.	Western blot BCA- kit (Thermo Scientific)
Al-Daghri, 2015	CSS	35 M/48 F diabetes type 2 patients and 42 M/39 F healthy	Habitual PA was positively associated with Irisin in healthy (r=0.20, p=0.03).	Irisin was positively correlated with waist circumference (r=0.23, p=0.04) in healthy and negatively with LBM (r=-0.26, p=0.02) and diastolic blood pressure (r=-0.25, p=0.02) in diabetes type II patients.	PP. (CNS)
Hofmann, 2014	CSS	39 anorexic F	Irisin was not correlated with numbers of steps per day.	No relationship of Irisin, with METs, energy expenditure.	PP, RK-067-16
Ijiri 2015b	CSS	65 M and seven F COPD patients. 24 M and three F healthy controls	Physical activity levels were positively associated with Irisin in both COPD patients (r=0.83, p<0.01) and healthy controls (r=0.79, p<0.001).	NA	PP, Burlingame. (CNS)
Kwaśniewska, 2016	CSS	62 healthy M	Irisin was positively correlated with physical activity levels in individuals demonstrated high weekly energy expenditure (2050–3840 kcal/week) (p=0.04).	Irisin was inversely correlated with VO2peak (p<0.05).	ELISA BioVendor, Czech Republic
Moreno, 2015	CSS	191 M and 230 F non-diabetic	Irisin was higher in physically active (128.55±78.71 ng/ml) than in sedentary individuals (105.66±60.2) (p=0.006).	Irisin was positively associated with weight (r=0.13, p=0.008), BMI (r=0.15, p=0.002), triglycerides (r=0.17, p<0.0001), insulin (r=0.11, p=0.020) and HOMA (r=0.10, p=0.037) and negatively with HDL (r=-0.19, p=0.001).	AB INC, Santa Clara, CA, SK00170-01
Palermo 2015	CSS	65 postmenopausal F affected by osteoporosis	No correlation between circulating Irisin and daily PA.	No relationship between Irisin and LBM, fat mass, body mass density and METs.	AG-45A- 0046EK-KI01; Adipogen AG, Liestal, Switzerland
Pardo, 2014	CSS	30 anorexic, 66 obese, 49 healthy F	Irisin was negatively correlated with daily PA (r=−0.22, p=0.001).	Irisin was positively correlated with REE (r=0.34, p=0.001), LBM (r=0.43, p=0.001), fat mass (r=0.52, p<0.001), glucose (r=0.22, p=0.0026), insulin (r=0.34, p<0.001), HOMA (r=0.33, p=0.001), BMI (r=0.52, p<0.001).	PP, EK-067-52
Jedrychowski 2015	CSS	Four sedentary controls M and Six young healthy M	Irisin was higher in exercisers (4.3 ng/ml) after a 12-week high-intensity aerobic CE compared to non-exercisers (3.6 ng/ml) (p=0.04).	NA	Mass spectrometer (Thermo Fisher Scientific)
**UCP1**					
***Chronic exercise***					
Norheim, 2014	CT	13 M healthy controls, and 11 M pre-diabetic	CE increased UCP1 mRNA in subcutaneous WAT (1.82-fold over baseline, p<0.05) when data of both groups were combined	NA	NA

C-RCT: cross-over randomized controlled trail; F: females; M: males; AE: Acute exercise; PGC-1α: peroxisome proliferator-activated receptor-γ coactivator 1α; FNDC5: Fibronectin type III domain-containing protein 5; PP: Phoenix Pharmaceuticals; NA: none available; CT: Controlled trial; CE: chronic exercise; UCP1: Uncoupling protein 1; WAT: White adipose tissue; CI: confidence interval; HOMA: homeostatic model assessment; CNS: Code not specified; SGS: Single group design studies; VO _2_max: Maximal oxygen uptake; CSS: Cross-sectional study; RCT: Randomized control trial; COPD: Chronic obstructive pulmonary disease; AB: Aviscera Bioscience; BMI: Body mass index; MetS: Metabolic Syndrome; LBM: Lean body mass; WHR: Waist to hip ratio; REE: Resting energy expenditure; VO2peak: peak oxygen uptake; WHR: waist to hip ratio; ATP: Adenosine triphosphate; PA: Physical activity; HDL: High density lipoprotein; METs: Metabolic equivalent.

### Risk of bias and quality of reporting data

The estimated risk of bias assessment results can be found in Table 2, and a summary is displayed in Supplementary Figure 2. Five RCTs ^44, 49– 52^, and all the included CTs and CSS, as well as 22 of the 23 SGS, displayed a high risk of bias due to inadequate generation of a randomised sequence, while four RCTs ^23, 33, 42, 53^ showed low risk of bias, and three RCTs ^41, 54, 55^, as well as one SGS ^56^, showed unclear risk of bias because there was no description of the method used for allocation (even though the participants were said to be “randomly” assigned). Six RCTs ^23, 42, 49, 50, 52, 53^ displayed low risk of bias for “allocation concealment”, while two ^44, 54^ showed unclear risk of bias because of the lack of description of the randomization allocation. Also, four RCTs ^33, 41, 51, 55^, and all the included CTs and SGS, as well as CSS, showed high risk of bias due to the lack of concealment of allocations before assignment. In “blinding of participants and personnel”, all RCTs, CTs, SGS and CSS displayed high risk of bias because the exercise interventions could not be blinded to the participants.

**Table 2.** Risk of bias assessment using the Cochrane Collaboration’s tool.

First author	Random sequence generation	Allocation concealment	Blinding of participants and researchers	Blinding of outcome assessment	Incomplete outcome data	Selective reporting	Other bias
RCTs							
Bang, 2014	**+**	**-**	**-**	**-**	**+**	**+**	**+**
Greulich, 2014	**+**	**+**	**-**	**+**	**+**	**+**	**+**
Greulich, 2014a	**+**	**+**	**-**	**?**	**+**	**+**	**+**
Hecksteden, 2013	**+**	**+**	**-**	**+**	**+**	**+**	**+**
Kim, 2015	**-**	**-**	**-**	**-**	**+**	**+**	**+**
Kim, 2016	**?**	**-**	**-**	**-**	**+**	**+**	**+**
Scharhag- Rosenberger, 2014	**?**	**?**	**-**	**+**	**+**	**+**	**+**
Huh, 2015	**-**	**?**	**-**	**?**	**?**	**+**	**+**
Nygaard, 2015	**-**	**+**	**-**	**?**	**?**	**+**	**+**
Tsuchiya, 2014	**-**	**+**	**-**	**?**	**?**	**+**	**+**
Tsuchiya, 2015	**-**	**+**	**-**	**?**	**?**	**+**	**+**
Tsuchiya, 2016	**?**	**-**	**-**	**-**	**?**	**+**	**+**
CTs							
Aydin, 2013	**-**	**-**	**-**	**-**	**?**	**+**	**+**
Ijiri, 2015a	**-**	**-**	**-**	**-**	**?**	**+**	**+**
Kraemer, 2014	**-**	**-**	**-**	**-**	**?**	**+**	**+**
Miyamoto-Mikami, 2015	**-**	**-**	**-**	**-**	**?**	**+**	**+**
Norheim, 2014	**-**	**-**	**-**	**?**	**?**	**+**	**+**
Pekkala, 2013	**-**	**-**	**-**	**-**	**?**	**+**	**+**
Prestes, 2015	**-**	**-**	**-**	**-**	**+**	**+**	**+**
Timmons, 2012	**-**	**-**	**-**	**-**	**?**	**+**	**+**
SGS							
Alvehus, 2014	**-**	**-**	**-**	**-**	**?**	**+**	**+**
Anastasilakis, 2014	**-**	**-**	**-**	**-**	**?**	**+**	**+**
Besse-Patin, 2014	**-**	**-**	**-**	**-**	**?**	**+**	**+**
Blüher, 2014	**-**	**-**	**-**	**-**	**+**	**+**	**+**
Boström, 2012	**-**	**-**	**-**	**-**	**?**	**+**	**+**
Camera, 2015	**-**	**-**	**-**	**-**	**?**	**+**	**+**
Comassi, 2015	**-**	**-**	**-**	**-**	**+**	**+**	**+**
Daskalopoulou, 2014	**-**	**-**	**-**	**-**	**?**	**+**	**+**
Ellefsen, 2014	**-**	**-**	**-**	**-**	**?**	**+**	**+**
Hew-Butler, 2015	**-**	**-**	**-**	**-**	**+**	**+**	**+**
Huh, 2012	**-**	**-**	**-**	**-**	**?**	**+**	**+**
Huh, 2014	**-**	**-**	**-**	**-**	**?**	**+**	**+**
Huh, 2014a	**-**	**-**	**-**	**-**	**?**	**+**	**+**
Khodadadi, 2014	**-**	**-**	**-**	**-**	**?**	**+**	**+**
Kurdiova, 2014	**-**	**-**	**-**	**-**	**?**	**+**	**+**
Lee, 2014	**-**	**-**	**-**	**-**	**?**	**+**	**+**
Löffler, 2015	**-**	**-**	**-**	**-**	**?**	**+**	**+**
Moraes, 2013	**-**	**-**	**-**	**-**	**?**	**+**	**+**
Murawska- Cialowicz, 2015	**-**	**-**	**-**	**-**	**+**	**+**	**+**
Moienneia, 2016	**?**	**-**	**-**	**-**	**+**	**+**	**+**
Palacios-González, 2015	**-**	**-**	**-**	**-**	**?**	**+**	**+**
Raschke, 2013	**-**	**-**	**-**	**-**	**?**	**+**	**+**
Scalzo, 2014	**-**	**-**	**-**	**-**	**?**	**+**	**+**
CSS							
Al-Daghri, 2015	**-**	**-**	**-**	**-**	**?**	**+**	**+**
Hofmann, 2014	**-**	**-**	**-**	**-**	**?**	**+**	**+**
Ijiri, 2015b	**-**	**-**	**-**	**-**	**?**	**+**	**+**
Jedrychowski, 2015	**-**	**-**	**-**	**-**	**?**	**+**	**+**
Kwaśniewska, 2016	**-**	**-**	**-**	**-**	**+**	**+**	**+**
Lecker, 2012	**-**	**-**	**-**	**-**	**?**	**+**	**+**
Moreno, 2015	**-**	**-**	**-**	**-**	**?**	**+**	**+**
Palermo, 2015	**-**	**-**	**-**	**-**	**?**	**+**	**+**
Pardo, 2014	**-**	**-**	**-**	**-**	**?**	**+**	**+**

**+**: Low risk of bias; **-**: High risk of bias; **?**: Unclear risk of bias; RCT: Randomised controlled trials; CT: Controlled trials; SGS: Single group design studies; CSS: Cross sectional studies.

In “blinding of outcome assessment”, three RCTs displayed low risk of bias ^23, 53, 54^, while five RCTs ^42, 44, 49, 50, 52^ and one CT ^16^ showed unclear risk of bias because of the lack of information regarding the blinding of assessments. Also, four RCTs ^33, 41, 51, 55^, the remaining seven CTs, and all the included SGS and CSS showed high risk of bias due to the knowledge of the allocated interventions by the assessors. Seven RCTs ^23, 33, 41, 42, 51, 53, 54^, one CT ^37^, five SGS ^39, 56– 59^ and one CSS ^60^ displayed low risk of bias, while five RCTs ^44, 49, 50, 52, 55^, the remaining seven CTs, the remaining 18 SGS and the remaining eight CSS showed unclear risk of bias for “incomplete outcome data” because of the lack of information on the participants who dropped out or exclusions in the analysis. All the included studies showed low risk of bias of “selective reporting” because they reported all the outcomes measured, and all the included studies displayed low risk of bias in “other bias”.

The results of our evaluation in the quality of the reporting data showed a mean score of 13.6 out of 25 (54.4%) for the included RCTs, 10.56 out of 18 (58.68%) for the included CTs and 10.52 out of 18 (58.44%) for the included SGS ( Table 3). The CSS displayed a mean score of 13.37 out of 22 (60.8%) ( Table 4). The score represents the number of items (with percentage of items) on the checklist that were reported satisfactorily in each study. Therefore, a high score represents a high adherence to reporting guidelines, while a low score represents low adherence to reporting guidelines.

**Table 3.** Quality of the reporting of the results using the CONSORT checklist. Score represents the number of items (with percentage of items) on the checklist that were reported satisfactorily in each study. Therefore, a high score represents a high adherence to reporting guidelines, while a low score represents low adherence to reporting guidelines.

	First author	Design	Score
1	Hecksteden, 2013	RCT	(16.5/25) 66%
2	Bang, 2014	RCT	(14.5/25) 58%
3	Greulich, 2014	RCT	(18/25) 72%
4	Greulich, 2014a	RCT	(16.5/25) 66%
5	Scharhag-Rosenberger, 2014	RCT	(15/25) 60%
6	Tsuchiya, 2014	RCT	(10.5/25) 42%
7	Nygaard, 2015	RCT	(11.5/25) 46%
8	Kim, 2015	RCT	(14/25) 56%
9	Kim, 2016	RCT	(14/25) 56%
10	Huh, 2015	RCT	(12.5/25) 50%
11	Tsuchiya, 2015	RCT	(12/25) 48%
12	Tsuchiya, 2016	RCT	(8.5/25) 34%
13	Timmons, 2012	CT	(6/18) 33%
14	Pekkala, 2013	CT	(9/18) 50%
15	Aydin, 2013	CT	(10.5/18) 58%
16	Norheim, 2014	CT	(11/18) 61%
17	Kraemer, 2014	CT	(10.5/18) 58%
18	Ijiri, 2015a	CT	(11.5/18) 64%
19	Miyamoto-Mikami, 2015	CT	(11.5/18) 64%
20	Prestes, 2015	CT	(14.5/18) 80%
21	Boström, 2012	SGS	(7/18) 39%
22	Huh, 2012	SGS	(10.5/18) 58%
23	Raschke, 2013	SGS	(9/18) 50%
24	Moraes, 2013	SGS	(12/18) 67%
25	Murawska-Cialowicz, 2015	SGS	(9.5/18) 52.7%
26	Moienneia, 2016	SGS	(9/18) 50%
27	Alvehus, 2014	SGS	(10/18) 55%
28	Besse-Patin, 2014	SGS	(13/18) 72%
29	Ellefsen, 2014	SGS	(10/18) 55%
30	Huh, 2014	SGS	(11/18) 61%
31	Kurdiova, 2014	SGS	(11.5/18) 64%
32	Scalzo, 2014	SGS	(11/18) 61%
33	Anastasilakis, 2014	SGS	(11.5/18) 64%
34	Blüher, 2014	SGS	(14/18) 80%
35	Daskalopoulou, 2014	SGS	(12.5/18) 69%
36	Huh, 2014a	SGS	(6/18) 33%
37	Khodadadi, 2014	SGS	(10.5/18) 58%
38	Lee, 2014	SGS	(8.5/18) 47%
39	Camera, 2015	SGS	(7/18) 39%
40	Comassi, 2015	SGS	(13/18) 72%
41	Hew-Butler, 2015	SGS	(12.5/18) 69%
42	Löffler, 2015	SGS	(11/18) 61%
43	Palacios-González, 2015	SGS	(12/18) 67%

CONSORT: Consolidated Standards of Reporting Trials; RCT: Randomized controlled trial; CT: Controlled trial; SGS: Single group design study.

**Table 4.** Quality of the reporting of the results using the STROBE checklist. Score represents the number of items (with percentage of items) on the checklist that were reported satisfactorily in each study. Therefore, a high score represents a high adherence to reporting guidelines, while a low score represents low adherence to reporting guidelines.

	First author	Design	Score
1	Lecker, 2012	CSS	(13.3/22) 60.45 %
2	Pardo, 2014	CSS	(12.2/22) 55.45 %
3	Hofmann, 2014	CSS	(15.6/22) 70.9 %
4	Ijiri, 2015b	CSS	(13.5/22) 61.36%
5	Jedrychowski 2015	CSS	(12/22) 54.45 %
6	Kwaśniewska, 2016	CSS	(15/22) 68.1 %
7	Moreno, 2015	CSS	(12.5/22) 56.81 %
8	Palermo, 2015	CSS	(13.5/22) 61.36 %
9	Al-Daghri, 2015	CSS	(12.8/22) 58.18 %

STROBE: Strengthening the Reporting of Observational Studies in Epidemiology; CSS: Cross-sectional study.

### Reporting of the outcomes

***The link between PGG-1a and FNDC5 in muscle in response to physical activity/exercise***

Acute effects of exercise

Five studies ^16, 17, 50, 61, 62^ investigating the link between PGC-1α with FNDC5 in muscle in response to acute exercise showed an increase of the PGC-1α mRNA in muscle; however, only two studies ^16, 62^ also found an increase in muscle FNDC5 mRNA, while one study ^43^ detected a positive association of PGC-1α with FNDC5 in muscle. More specifically, a study found that an aerobic (2.1±0.8-fold over baseline, p=0.05) and a resistance (3.5±0.9-fold over baseline, p=0.01) training session increased PGC-1α splice variant1 but it did not change FNDC5 mRNA in the muscle of healthy adults ^50^. Similarly, a resistance training session increased PGC-1α splice variant1 four hours post exercise (200%, over baseline and over control, p<0.05), but it did not change FNDC5 mRNA in the muscle of healthy adults ^61^. A 45-minute endurance exercise session increased Exon 11 of PGC-1α mRNA in muscle (7.4-fold over baseline, p<0.05), but it did not change FNDC5 mRNA in muscle in both healthy and pre-diabetic adults, while a positive association between PGC-1α and FNDC5 mRNA was found at baseline (r=0.82, p<0.01) when data of the two groups were combined ^16^. Furthermore, PGC-1α mRNA in muscle increased (>6-fold over baseline, p<0.05) in response to acute exercise; however, FNDC5 mRNA in muscle was not altered in sedentary overweight and obese adults ^17^. Also, a resistance exercise session increased Exon 11 of PGC-1α mRNA in muscle of both young (4-fold over baseline, p<0.05) and older (2-fold over baseline, p<0.05) healthy adults, while it increased FNDC5 mRNA in muscle only in young (1.4-fold over baseline, 95% Confidence Interval=0.3–2.2, p<0.05) healthy adults ^62^. Finally, PGC-1α mRNA in muscle was positively associated with FNDC5 mRNA in muscle (r=0.56, p<0.05) in a sub-set of 24 patients with heart failure ^43^; stratification was *ad hoc*.

Chronic effects of exercise

Of the eleven eligible studies ^2, 16– 19, 40, 62– 67^ that examined the link between PGC-1α with FNDC5 in muscle in response to chronic exercise, only two ^16, 66^ showed that chronic exercise increased PGC-1α and FNDC5 mRNA in muscle, while four studies ^18, 19, 62, 63^ showed no effect of chronic exercise on PGC-1α and FNDC5 mRNA in muscle. In the five studies that only measured FNDC5 in muscle, one study ^2^ found increased and four ^17, 40, 64, 65^ showed no effect of chronic exercise on FNDC5 mRNA in muscle.

A 12-week of endurance and resistance combined exercise training increased Exon 11 of PGC-1α mRNA in muscle (1.2-fold in healthy and 1.6-fold in pre-diabetic adults over baseline, p<0.05) and FNDC5 mRNA in muscle (1.4-fold in healthy and 2-fold in pre-diabetic adults over baseline, p<0.05) ^16^. Furthermore, an 8-week sprints exercise program increased PGC-1a and FNDC5 mRNA in muscle (p<0.05) in healthy adults ^66^. Finally, Bostrom *et al.* (2012) showed that in eight older participants selected from a larger group of 27 participants, chronic exercise increased FNDC5 mRNA in muscle (p<0.05) ^2^.

A 21-week endurance and resistance combined exercise program in healthy adults did not alter PGC-1α and FNDC5 mRNA in muscle ^62^. One of the included studies ^19^ found no effect of chronic exercise on PGC1a or FNDC5 mRNA in younger adults (despite detecting significant changes in ~1,000 other mRNAs and finding mitochondrial enzyme activity was increased in ~25%) ^68^. Similarly, an 8-week resistance exercise program did not alter PGC-1α or FNDC5 mRNA in muscle of young healthy adults ^63^. In addition, 12 weeks of resistance training did not alter PGC-1α splice variant1 mRNA, and it did not change the FNDC5 mRNA in muscle in untrained young females ^18^. Also, a 12-week aerobic and resistance exercise combined program ^17^ and an 8-week aerobic exercise program ^40^ did not alter FNDC5 mRNA in muscle of sedentary obese adults, while chronic exercise had no effect on FNDC5 mRNA in muscle of healthy adults ^64^. Finally, a 3-week sprint interval training program did not alter FNDC5 mRNA in muscle of healthy adults ^65^. 

***The effects of physical activity/exercise on Irisin***

Acute effects of exercise

Studies using enzyme-linked immunosorbent assays (ELISA)

Eighteen of the included studies ^12^, ^17^, ^21^, ^22^, ^34^, ^35^, ^38^, ^44^, ^49^, ^50^, ^52^, ^56^, ^58^, ^62^, ^66^, ^69– 71^ examined the effects of acute exercise on circulating Irisin, and a further seven studies ^34, 36, 46– 48, 60, 72^ investigated the association of circulating Irisin with physical activity levels using commercial ELISA kits. Thirteen studies ^12, 21, 22, 38, 44, 49, 50, 52, 58, 66, 69– 71^ showed that acute exercise increased circulating Irisin in healthy individuals, while five studies ^17, 34, 35, 56, 62^ showed no effect of acute exercise on circulating Irisin. Also, three studies ^34, 48, 60^ showed a positive association of circulating Irisin with physical activity levels in healthy and COPD patients, while four studies ^36, 46, 47, 72^ showed no association or a negative association of circulating Irisin with physical activity levels in both healthy and clinical populations.

A resistance training session did not change FNDC5 mRNA in the muscle of healthy adults and circulating Irisin increased (p<0.001) over the following 24-hour ^50^, indicating no short-term association between FNDC5 and Irisin. Furthermore, an aerobic exercise session increased circulating Irisin (p=0.04) and Irisin concentrations were measured at ~355–459 ng/ml ^50^, greater than recent mass spectrometry measurements ^73^. Similarly, a running exercise session in healthy individuals ^49^ and an aerobic exercise session, as well as a resistance exercise session, in healthy individuals and in metabolic syndrome patients ^44^ increased circulating Irisin (p<0.05). In the latter studies, Irisin concentrations measured at ~99–175 ng/ml ^49^ and ~80–94.6 ng/ml ^44^, respectively, which is greater than recent mass spectrometry measurements ^73^. Also, an acute resistance exercise session increased circulating Irisin (p<0.05) as oppose to aerobic and combined (aerobic and resistance) sessions that did not alter circulating Irisin in healthy males (Irisin concentrations ~18–151 ng/ml) ^52^. Furthermore, a 90-minute aerobic exercise session increased circulating Irisin during (54 ^th^ minute) the exercise session (20.4% compared to baseline, F _(3,36)_=5.28, p=0.004), but circulating Irisin decreased after the exercise session (p=0.021) in healthy male adults ^69^. In the latter study, the aerobic exercise session also increased circulating Irisin during (54 ^th^ minute) the exercise session (F _(3,24)_=5.03, p=0.01) in healthy female adults ^69^. Eight out of the 23 included SGS showed that acute exercise increased circulating Irisin in healthy populations ^12, 21, 22, 38, 58, 66, 70, 71^, while a resistance exercise session increased FNDC5 mRNA in muscle only in young healthy adults and it did not alter circulating Irisin of both young and older healthy adults ^62^. In addition, 45 minutes of running did not alter circulating Irisin in obese healthy adults ^35^. Similarly, an acute cycling session did not alter circulating Irisin in COPD patients ^34^, while an acute exercise session did not alter FNDC5 mRNA in muscle or circulating Irisin in sedentary overweight and obese adults ^17^. Finally, an acute exercise session of both low and high intensity resistance training did not alter circulating Irisin (p>0.05) in sedentary young healthy females (Irisin concentrations ~69–87 ng/ml) ^56^.

Physical activity levels were positively associated with circulating Irisin in healthy adults (r=0.20, p=0.03), but not in patients with diabetes type II ^48^, and they were not associated with circulating Irisin in osteoporotic women ^46^ and in anorexic women ^47^. Furthermore, circulating Irisin concentrations were higher in physically active (Irisin concentrations 128.55±78.71 ng/ml) than in sedentary individuals (Irisin concentrations 105.66±60.2 ng/ml) (p=0.006) ^72^. However, physical activity levels were negatively associated with circulating Irisin (r=−0.22, p=0.001) in groups of anorexic, obese and healthy women ^36^, while they were positively associated with circulating Irisin in both COPD patients (r=0.83, p<0.01) and healthy individuals (r=0.79, p<0.001) ^34^. Finally, circulating Irisin was positively correlated with physical activity levels in individuals who demonstrated high weekly physical activity energy expenditure (2050–3840 kcal/week) (Irisin concentrations ~32–261 ng/ml, p=0.04).

Studies using mass spectrometry and western blotting

Only one included study used both western blotting and mass spectrometry to detect circulating Irisin in response to acute exercise. This study showed that submaximal acute aerobic exercise increased circulating Irisin (3.1-fold over baseline, p<0.05), whereas maximal acute aerobic exercise did not alter circulating Irisin, even though tended to be significant (p=0.07), in two healthy volunteered adults ^24^.

Chronic effects of exercise

Studies using ELISA

Twenty three included studies ^12, 16, 18, 22, 23, 33, 34, 37, 39, 41, 42, 45, 51, 53– 57, 59, 62, 65, 70, 74^ in the current review examined the effects of chronic exercise on circulating Irisin using commercial ELISA kits, while the populations examined showed large heterogeneity. Nine studies ^23, 34, 39, 41, 51, 54, 59, 65, 74^ showed that chronic exercise increased circulating Irisin, while 12 studies ^12, 16, 18, 22, 33, 37, 42, 45, 53, 57, 62, 70^ showed no effects of chronic exercise on circulating Irisin, and two studies showed that chronic exercise decreased circulating Irisin ^55, 56^, in both healthy and clinical populations.

A 6-month resistance training program increased circulating Irisin in healthy controls (p<0.01), but not in the exercisers ^54^, while an 8-day vibration exercise increased circulating Irisin in COPD patients (p=0.01) ^23^. Notably, the Irisin concentrations in the latter study ^23^ were ~785–1196 ng/ml, a lot greater than recent mass spectrometry based detection of Irisin concentrations ^73^. Furthermore, a 12-week resistance exercise increased circulating Irisin in elderly healthy females (Irisin concentrations ~61–83 ng/ml, p<0.05,) ^51^. In addition, a 12-week of endurance and resistance combined exercise training in both healthy and pre-diabetic adults increased FNDC5 mRNA in muscle, while it decreased circulating Irisin (p<0.05) when the data of both healthy and pre-diabetic groups were combined ^16^. In the latter study, Irisin concentrations were detected at 160 ng/ml at baseline and 143 ng/ml after the exercise program, a lot greater than recent mass spectrometry based detection of Irisin concentrations ^73^. In addition, an 8-week endurance training program increased circulating Irisin only in middle-aged and not in young healthy adults (Irisin concentrations ~140–168 ng/ml, p<0.05) ^74^, while an 8-week chronic exercise program in COPD patients increased circulating Irisin (p<0.05) ^34^. Finally, a 12-month physical activity intervention increased circulating Irisin by ~12% (p=0.001) in obese children ^39^. Notably, in the latter study, Irisin concentrations were 111 ng/ml, a lot greater than recent mass spectrometry based detection of Irisin concentrations ^73^.

A 3-week sprint interval training program did not alter FNDC5 mRNA in muscle and showed a gender difference in circulating Irisin, which was decreased in healthy males and increased in healthy females (p<0.05) ^65^. An 8-week resistance exercise training program increased circulating Irisin compared to control group (p<0.05), while the Irisin concentrations were ~700–850 ng/ml ^41^. Similarly, 3-month cross-fit training increased circulating Irisin (Irisin concentrations ~300–850 ng/ml, p<0.05) only in females ^59^. On the other hand, a 4-week sprint exercise training decreased circulating Irisin (Irisin concentrations ~200-340 ng/ml, p<0.05) in healthy males ^55^. Three months of both non-individualized training and individualized training did not alter circulating Irisin (Irisin concentrations ~123–131 ng/ml, p>0.05) in COPD patients ^42^. Finally, an 8-week low intensity resistance training program did not alter circulating Irisin, while an 8-week high intensity resistance training program reduced circulating Irisin (Irisin concentrations ~51–87 ng/ml, p=0.03) ^56^.

An 8-week resistance training program in healthy adults did not alter circulating Irisin ^33^ and a 26-week aerobic exercise program revealed no changes in circulating Irisin of healthy adults ^53^. A 21-week endurance and resistance combined exercise program in healthy adults did not alter FNDC5 mRNA in muscle and circulating Irisin ^62^. Similarly, a 16-week resistance exercise program in elderly women did not increase circulating Irisin ^37^ and 12 weeks of resistance training did not alter FNDC5 mRNA in muscle or circulating Irisin ^18^. However, circulating Irisin was positively correlated with FNDC5 mRNA in muscle (r=0.65, 95% Confidence Interval=0.12–0.89, p<0.05) in the latter study ^18^. Finally, five SGS showed that chronic exercise did not alter circulating Irisin in healthy individuals ^12, 22, 57, 70^ and haemodialysis patients ^45^.

Studies using mass spectrometry and western blotting

Only two included studies used alternative methods than commercial ELISA kits to detect human circulating Irisin in response to chronic exercise. Initially, Bostrom *et al*. (2012) showed via western blotting that in eight older participants selected from a larger group of 27 participants ^67^ chronic exercise increased FNDC5 mRNA in muscle (p<0.05) and circulating Irisin (2-fold over baseline, p<0.05) ^2^. Finally, one study contrasted plasma Irisin concentrations in six younger individuals following 12 weeks high intensity aerobic exercise with those found in a separate group of four individuals (no pre-training samples were presented) ^73^. This study used mass spectrometry and detected circulating Irisin at 3.6 ng/ml in controls and 4.3 ng/ml in exercisers, which was significantly different between the two groups (p=0.04). No details regarding training or control of hydration in the training group were reported ^73^.

### The effects of physical activity/exercise on UCP1 in WAT

We located only one study that examined the effects of exercise on UCP1 mRNA in subcutaneous WAT in humans. This study found that a 12-week intervention of endurance and resistance combined exercise in both healthy and pre-diabetic adults had no significant effect on UCP1 mRNA in subcutaneous WAT, even though UCP1 mRNA was increased (1.82-fold over baseline, p<0.05) when data from both groups were combined ^16^. Also, UCP1 mRNA did not associate with FNDC5 mRNA in muscle (r=0.28, p=0.18) and circulating Irisin (r=-0.11, p=0.60) ^16^.

### Results for associations of Irisin with secondary outcome measures

The secondary results of the included studies can be found in Table 1. In 118 muscle profiles, FNDC5 mRNA was modestly and positively correlated with BMI (r ^2^=0.1, p=0.004), while FDNC5 mRNA was not related to fasting glucose or glycaemic control ^19^. Furthermore, circulating Irisin was not associated with inflammatory indices ^39^, blood glucose ^62, 65^, homeostatic model assessment (HOMA) ^62, 65, 71^, insulin ^62, 65, 71^, leptin ^71^, lean body mass ^46, 57^, fat mass ^18, 46, 57^, waist to hip ratio ^71^, energy expenditure ^21, 54^, BMI ^71^, and pulmonary function ^34^.

Additional secondary results show that circulating Irisin was positively associated with BMI ^59, 70, 72, 75^, triglycerides ^70, 72^, fat mass ^36, 59^, HOMA ^72^, insulin ^72^, blood glucose ^71^ and leptin ^75^, and negatively with high density lipoprotein cholesterol ^70^, all of which indicate unfavourable effects of Irisin on human health. Nevertheless, some secondary evidence suggests that circulating Irisin was positively associated with fat free mass ^36, 70^, muscle mass ^41^ and energy expenditure ^36^, and Irisin that was incubated within white adipocytes *in vitro* increased glucose and fatty acids uptake ^66^. Furthermore, circulating Irisin after a maximal workload was significantly greater in individuals with higher VO _2_max than individuals with lower VO _2_max ^21^. However, circulating Irisin was not associated with VO _2_peak before and post exercise in healthy females ^57^ and sedentary overweight and obese individuals, while it was inversely correlated with VO _2_peak (p<0.05) in healthy males ^60^.

## Discussion

The aim of the current review was to systematically identify the effects of physical activity on the link between PGC-1a and FNDC5 in muscle and circulating Irisin, as well as evidence for regulation of UCP1 in WAT (indicating a browning process) in humans.

### Overall completeness and applicability of evidence

We were unable to find strong evidence that links PGC-1α and FNDC5 mRNA in muscle in response to exercise training or increased physical activity levels. Notably, we located only one study that examined the effects of exercise on UCP1 in WAT, and this found no effect ^16^. Despite PGC-1α being firmly placed as a central regulator of adaptation to exercise in mice and humans, numerous aspects of the literature are contradictory or incomplete. For example, previous evidence indicates that PGC-1α mRNA accumulates with endurance training, while studies of PGC-1α protein reflect various antibodies that measure distinct molecular entities ranging from 70 to >110 kDa ^76– 78^. Furthermore, mice lacking PGC-1α adapt normally to endurance exercise training, and in humans the PGC-1α regulated gene network does not correlate with aerobic adaptation ^68^. Thus any argument that places Irisin as part of the core PGC-1α regulated exercise adaptation program needs to reflect, on both technical and theoretical grounds, that there is great uncertainty of the nature and importance of PGC-1α in exercise and health ^79^.

When PGC-1α protein content is measured (albeit with uncertainty over protein identities) exercise training increases PGC-1α protein in skeletal muscle or causes nuclear translocation of protein ^80– 83^. However, the studies included in the current review only relied on measuring PGC-1α mRNA to determine the effects of exercise on PGC-1α, and the time-course of mRNA and protein responses to exercise are distinct. Thus, the link between PGC-1α and FNDC5 in skeletal muscle may reflect measurement of mRNA dynamics and this may explain inconsistent findings for PGC-1α. Also, the proposed mechanism by Bostrom *et al*. (2012) indicates that induction of PGC-1α mRNA and then protein would activate the transcription of FNDC5, and hence, if this theory was correct, it would be expected that a strong correlation between PGC-1α mRNA and FNDC5 mRNA would exist. However, previous evidence showed that FNDC5 mRNA in muscle is not regularly increased by exercise or differently regulated between those with and without insulin resistance ^19^, and was only modestly increased in a subset of older people following chronic exercise training ^19^. If we focus on more reliable mRNA measures of PGC-1α and FDNC5, then the variable findings may be explained by the different characteristics of the populations examined and the different exercise protocols used.

 An interesting aspect brought forward in the included studies showed that the start codon of the FNDC5 gene displays a variation in humans due to the non-ATG start codon ^64^. In humans, ATG is usually the first codon to lead to efficient protein production, and therefore, the latter may suggest that Irisin, if produced, would be done so in an inefficient manner ^64^. However, this notion has been questioned by a subsequent study, which supports that human Irisin is mainly translated from its non-ATG start codon, while the molecular weight of the protein is similar to that of important proteins in human body, such as insulin, leptin and resistin ^73^, indicating a biological role of Irisin.

The various commercially available antibodies used in the ELISA kits of the studies included in the current systematic review, yield a protein concentration that appears to be ~5–278 times greater than a more recent mass spectrometry data (data that may require independent validation), and still far above what others have found ^84^. Furthermore, Kurdiova *et al.* (2014) reported poor agreement between ELISA kit RK-067-16 and EK-067-29 (Phoenix Pharmaceuticals) ^17^. Similarly, no correlation was found between EK-067-52 and ELISA of Adipogen that were used in the same samples ^26^. Finally, Montes-Nieto *et al.* (2016) analysed human Irisin using two different lots (604824 and 605835) of the ELISA kit EK-067-29 (Phoenix Pharmaceuticals) and also found a poor agreement (r=0.226) between them ^85^. These technical considerations may explain part or all of the equivocal results of the included studies in this current review regarding circulating Irisin.

According to the results of the current systematic review, two studies have measured circulating Irisin via mass spectrometry in response to exercise in humans. In the study by Jedrychowski *et al*. (2015), blood samples for Irisin identification were collected only after the exercise program from a small number of participants who were sedentary (n=4) or aerobic exercisers (n=6) ^73^. In the study by *Lee et al*. (2014), Irisin was measured only pre and post-acute exercise without a control situation, and the sample size was only two participants ^24^. Also, in the latter study a ~3-fold increase of Irisin was reported only after submaximal and not maximal exercise. These studies display methodological limitations and a small number of participants, which indicates that future longitudinal studies of changes in Irisin will clarify if the mass spectrometry measures reflect exercise-induced changes. Furthermore, Bostrom *et al.* (2012) and Lee *et al.* (2014) used an antibody that is discontinued for Irisin identification, given that it recognises a peptide of FNDC5 that is not part of the sequence of the secreted Irisin as this identified by mass spectrometry ^73^, while Jedrychowski *et al.* (2015) used an antibody by Adipogen. This may explain the discrepancy in the molecular weight of Irisin between those analysed by Bostrom *et al.* (2012) and Lee *et al.* (2014) (~22 kDa) and those analysed by Jedrychowski *et al.* (2015) (~12 kDa). While the studies that utilised mass spectrometry do not agree ^24, 73^, reflecting issues of sensitivity and methodology, the latest identification and analysis of Irisin ^24, 73^ indicates that Irisin may circulate in blood and probably has a similar or identical structure to the mouse structure; however, whether it has genuine biological activity remains to be elucidated.

### Quality of evidence and limitations

Based on the studies selected for the purposes of the current review, we cannot reach precise conclusions regarding the effects of acute and chronic exercise on PGC-1α in conjunction with FNDC5 mRNA in muscle; this is mainly due to the inconsistency of the findings and the different population characteristics examined. Most of the RCTs ^33, 44, 49– 52^ display high risk of bias, due to inadequate generation of a randomised sequence and a lack of concealment of allocations before assignment, while all the RCTs exhibit high risk of bias since the exercise interventions could not be blinded to the participants. In addition, four RCTs ^44, 49, 50, 52^ display unclear risk of bias because of the lack of information regarding the blinding procedures. Therefore, the risk of bias assessment of the included RCTs indicates that they may provide imprecise results ( Table 2). In addition, the CTs and SGS display a high risk of bias due to the absence of generation of a randomised sequence, inadequate concealment of allocations before assignment and knowledge of the allocated interventions by the outcome assessors. They also display unclear risk of bias due to knowledge of the allocated interventions by the investigators during the study ( Table 2). Finally, the included CSS display high risk of bias due to inadequate generation of a randomised sequence, lack of concealment of allocations before assignment and knowledge of the allocated interventions by the assessors, while they display unclear risk of bias for “incomplete outcome data” because of the lack of information of the participants who were excluded from the analysis. This evidence indicates that the CTs, SGS and CSS may also provide imprecise results. Furthermore, quality of reporting, as expressed through the adherence guidelines (i.e. CONSORT and STROBE), showed low scores of the required results that should have been reported (54.4% for RCTs, 58.68% for CTs, 58.44% for SGS and 60.8% for CSS) by the included studies in the current review. This shows inadequate reporting of the results of the included studies that may not aid the critical appraisal and interpretation of their outcomes.

### Agreements and disagreements with other studies or reviews

To the best of our knowledge, this is the first systematic review that examines the effects of physical activity on the link between PGC-1α and FNDC5 in muscle, circulating Irisin and on UCP1 of WAT in humans. We compared our results with a recent meta-analysis that aimed to identify the effects of exercise on circulating Irisin ^86^. This meta-analysis concluded that chronic exercise may decrease circulating Irisin in the RCTs while the non-RCTs cannot form any conclusion. However, the latter meta-analysis did not take into consideration the issues raised regarding the validity of the methods used for Irisin identification ^26^. In contrast, while we considered the methods used for Irisin identification in the studies included in the current review, our review had a different aim, to systematically identify the effects of physical activity on the link between PGC-1α and FNDC5 in muscle, circulating Irisin and find evidence for regulation of UCP1 in WAT in humans. Regarding circulating Irisin, we also report that we cannot form any firm conclusion of the effects of exercise on circulating Irisin. Our review highlights previous evidence showing that circulating Irisin may only be detected in humans via mass spectrometry ^25, 26, 73^, while we suggest that the previous available data coming from methods that have not been previously validated for circulating Irisin identification should not be used. This is because recent evidence questioned the antibodies used in the commercial ELISA kits given the polyclonal nature of these antibodies that may attract cross-reacting proteins ^26^. However, publications that use commercial ELISA that have not been previously validated to detect human Irisin continue at an alarming rate. Therefore, our review indicates to consider using only valid methods for human circulating Irisin identification in the future. Furthermore, our results are in accordance with a previous review that showed equivocal results among studies examining circulating Irisin due to the methodological variations for Irisin detection ^87^. In this critical review, the authors examined the commercial antibodies and ELISA used to measure circulating Irisin and concluded that the currently available antibodies should be tested for cross-reacting antigens detection ^87^. Additionally, another recent review showed that the previous measurements for circulating Irisin identification differs greatly, given that they displayed a molecular weight of the protein between 0.01 ng/ml and 2000 ng/ml ^88^. The latter critical review concluded that it is necessary to establish accurate methods for irisin measurements. Our systematic review analysis, agrees with the latter conclusion given that the Irisin measurements in the included studies via commercial ELISA kits, displayed a molecular weight of the protein ranging between 22 ng/ml to 1196 ng/ml.

Initially, Irisin was proposed to have a therapeutic effect given the potential to cause a browning formation of WAT that may have anti-obesity and antidiabetic effects ^2^. This was mainly suggested when Irisin administered in obese mice improved glucose homeostasis and caused weight loss ^2^. Also, the browning formation that Irisin may cause could lead to reduced weight gain, up-regulated insulin sensitivity, reduced risk of diabetes type II and other metabolic disorders as animal studies indicate ^89– 93^, as well as increase daily resting energy expenditure in humans ^94, 95^. The available evidence from the included studies in the current review revealed that the available commercial ELISA kits for Irisin identification either were found to be invalid ^26, 87^ or they should be tested for validity ^87^. Thus, we cannot confirm a favourable effect of Irisin on human metabolism. Finally, none of the included studies in the current review examined associations of circulating Irisin with indices indicate a therapeutic role of the protein using western blotting and/or mass spectrometry methods.

### Potential biases in the review process

The current review has a number of strengths. For instance, we used the PubMed and the EMBASE databases using appropriate algorithms with standardized indexing terms. Standardized indexing terms can retrieve records that may use different words to describe the same concept and information beyond that may be contained in the words of the title and abstract ^96^. Furthermore, the current review used a systematic manner to identify articles according to previous methodology ^27– 29^, and we used well-established tools ^30– 32^ to evaluate the included studies. To reduce bias, two investigators worked independently on the screening of the included studies for eligibility, risk of bias assessment, and in the provision of CONSORT and STROBE scores. Also, we have not excluded studies based on language. However, a limitation of the current review includes the use of only published literature; we did not include grey literature searching. In this light, there is a potential of publication bias in the current review. Nevertheless, the inclusion of grey literature may itself introduce bias and one reason to include grey literature would be the absence of peer-review sources ^96^.

## Conclusions

We found little evidence to determine the link between PGC-1a mRNA and FNDC5 mRNA in human muscle, and there was limited evidence on the effects of physical activity on UCP1 in subcutaneous WAT. We also found a heterogeneity in the populations examined, high risk of bias by the selected studies and a relatively small number of RCTs (n=12) with inconsistent findings regarding the link between physical activity, PGC-1a, FNDC5, and UCP1.

Mass spectrometry detection of Irisin of exercise effects were compromised by the methodological limitations of the existed studies (i.e. post exercise comparisons, lack of control, small samples). The current systematic review highlights previous evidence that indicates via mass spectrometry that Irisin is present in human blood at concentrations that are ~5–278 folds lower than those detected by commercial ELISA kits. Therefore, we are unable to conclude on the circulating Irisin response to physical activity due to methodological limitations. In this regard, our systematic review used well-established methodology (i.e. PRISMA and Cochrane Library guidelines). However, we have also considered the validity and accuracy of the measurements of Irisin protein concentrations in the included studies. This additional analysis completely redirected our conclusion compared to the conclusion that a well-established systematic review methodology would provide. Therefore, we suggest that future systematic reviews should also take into consideration the validity and accuracy of the measurements of the included studies, to avoid misleading conclusions. We also suggest that future studies should only consider currently valid methods for human circulating Irisin (i.e. mass spectrometry), until new methods are introduced. The latter also implies that future studies should re-examine the biological role for human Irisin and the effects of physical activity/exercise on the link between PGC-1a and FNDC5 in muscle, circulating Irisin and UCP1 in WAT.

## Supplementary material

**Supplementary File 1:** PubMed search.

.

**Supplementary File 2:** EMBASE search.

.

**Supplementary Table 1:** PRISMA checklist.

.

**Supplementary Figure 1:** PRISMA flow diagram of study selection and identification.

.

**Supplementary Figure 2:** Summary of risk of bias assessment using the Cochrane Collaboration’s tool.
