# Supplementary material for: Effects of physical activity on the link between PGC-1a and FNDC5 in muscle, circulating Ιrisin and UCP1 of white adipocytes in humans: A systematic review
Source: F1000Res. 2017 May 26;6:286. Originally published 2017 Mar 17. [Version 2] doi: 10.12688/f1000research.11107.2 (PMC5461915; doi:10.12688/f1000research.11107.2)
Supplement: Supplementary file 1 [file f1000research-6-12649-s0000.tgz › 95a7edb6-e18b-42e8-b3d2-09f741f32b1d.docx]

Petros C. Dinas, Ian M. Lahart, James A. Timmons, Per-Arne Svensson, Yiannis Koutedakis, Andreas D. Flouris, and George S. Metsios

**Effects of physical activity on the link between PGC-1a and FNDC5 in muscle, circulating irisin, and UCP1 of white adipocytes in humans: A systematic review**

## Supplementary File 1: PubMed search

1. PGC1 alpha[Title/Abstract]
2. PGC-1 alpha[Title/Abstract]
3. PGC-1a[Title/Abstract]
4. PGC1a[Title/Abstract]
5. PGC-1alpha[Title/Abstract]
6. Peroxisome proliferator-activated receptor gamma coactivator 1-alpha[Title/Abstract]
7. Peroxisome proliferator-activated receptor gamma coactivator 1-a[Title/Abstract]
8. Peroxisome proliferator-activated receptor gamma coactivator 1 alpha[Title/Abstract]
9. Peroxisome proliferator-activated receptor gamma coactivator 1a[Title/Abstract]
10. Peroxisome proliferator-activated receptor gamma coactivator 1alpha[Title/Abstract]
11. PGC1alpha[Title/Abstract]
12. (#1 OR #2 OR #3 OR #4 OR #5 OR #6 OR #7 OR #8 OR #9 OR #10 OR #11)
13. FNDC5[Title/Abstract]
14. Fibronectin type III domain-containing protein 5[Title/Abstract]
15. Irisin-encoding gene[Title/Abstract]
16. (#13 OR #14 OR #15)
17. Irisin[Title/Abstract]
18. PGC1alpha-dependent myokine[Title/Abstract]
19. (#17 OR #18)
20. Uncoupling protein one[Title/Abstract]
21. Uncoupling protein 1[Title/Abstract]
22. UCP1[Title/Abstract]
23. (#20 OR #21 OR #22)
24. (#12 OR #16 OR #19 OR #23)
25. exercise[MeSH Terms]
26. exercise[Title/Abstract]
27. motor activity[MeSH Terms]
28. sports[MeSH Terms]
29. sport*[Title/Abstract]
30. resistance training[MeSH Terms]
31. training[Title/Abstract]
32. fitness[Title/Abstract]
33. physical activity[Title/Abstract]
34. physical activities[Title/Abstract]
35. physical activity intervention*[Title/Abstract]
36. exercise intervention*[Title/Abstract]
37. active[Title/Abstract]
38. aerobic[Title/Abstract]
39. (#25 OR #26 OR #27 OR #28 OR #29 OR #30 OR #31 OR #32 OR #33 OR #34 OR #35 OR #36 OR #37 OR #38)
40. (#24 AND #39)
41. animals[MeSH Terms]
42. humans[MeSH Terms]
43. (#41 NOT #42)
44. (#40 NOT #43)
