# Supplementary material for: Effects of physical activity on the link between PGC-1a and FNDC5 in muscle, circulating Ιrisin and UCP1 of white adipocytes in humans: A systematic review
Source: F1000Res. 2017 May 26;6:286. Originally published 2017 Mar 17. [Version 2] doi: 10.12688/f1000research.11107.2 (PMC5461915; doi:10.12688/f1000research.11107.2)
Supplement: Supplementary file 2 [file f1000research-6-12649-s0001.tgz › ea61a67b-6c62-4ccc-8915-bff49b5f0eb0.docx]

Petros C. Dinas, Ian M. Lahart, James A. Timmons, Per-Arne Svensson, Yiannis Koutedakis, Andreas D. Flouris, and George S. Metsios

**Effects of physical activity on the link between PGC-1a and FNDC5 in muscle, circulating irisin, and UCP1 of white adipocytes in humans: A systematic review**

## Supplementary File 2: EMBASE search

1. PGC1 alpha.ti,ab.
2. PGC-1 alpha.ti,ab.
3. PGC-1a.ti,ab.
4. PGC1a.ti,ab.
5. PGC-1alpha.ti,ab.
6. Peroxisome proliferator-activated receptor gamma coactivator 1-alpha.ti,ab.
7. Peroxisome proliferator-activated receptor gamma coactivator 1-a.ti,ab.
8. Peroxisome proliferator-activated receptor gamma coactivator 1 alpha.ti,ab.
9. Peroxisome proliferator-activated receptor gamma coactivator 1a.ti,ab.
10. Peroxisome proliferator-activated receptor gamma coactivator 1alpha.ti,ab.
11. PGC1alpha.ti,ab.
12. 1 or 2 or 3 or 4 or 5 or 6 or 7 or 8 or 9 or 10 or 11
13. FNDC5.ti,ab.
14. Fibronectin type III domain-containing protein 5.ti,ab.
15. Irisin-encoding gene.ti,ab.
16. 13 or 14 OR 15
17. Irisin.ti,ab.
18. PGC1alpha-dependent myokine.ti,ab.
19. 17 or 18
20. Uncoupling protein one.ti,ab.
21. Uncoupling protein 1.ti,ab.
22. UCP1.ti,ab.
23. 20 or 21 or 22
24. 12 or 16 or 19 or 23
25. exercise/
26. exercise.ti,ab.
27. motor activity/
28. sport/
29. sport*.ti,ab.
30. resistance training/
31. training.ti,ab.
32. fitness.ti,ab.
33. physical activity.ti,ab.
34. physical activities.ti,ab.
35. physical activity intervention*.ti,ab.
36. exercise intervention*.ti,ab.
37. active.ti,ab.
38. aerobic.ti,ab.
39. 25 or 26 or 27 or 28 or 29 or 30 or 31 or 32 or 33 or 34 or 35 or 36 or 37 or 38
40. 24 and 39
