# Supplementary figures and images for: Effects of physical activity on the link between PGC-1a and FNDC5 in muscle, circulating Ιrisin and UCP1 of white adipocytes in humans: A systematic review
Source: F1000Res. 2017 May 26;6:286. Originally published 2017 Mar 17. [Version 2] doi: 10.12688/f1000research.11107.2 (PMC5461915; doi:10.12688/f1000research.11107.2)

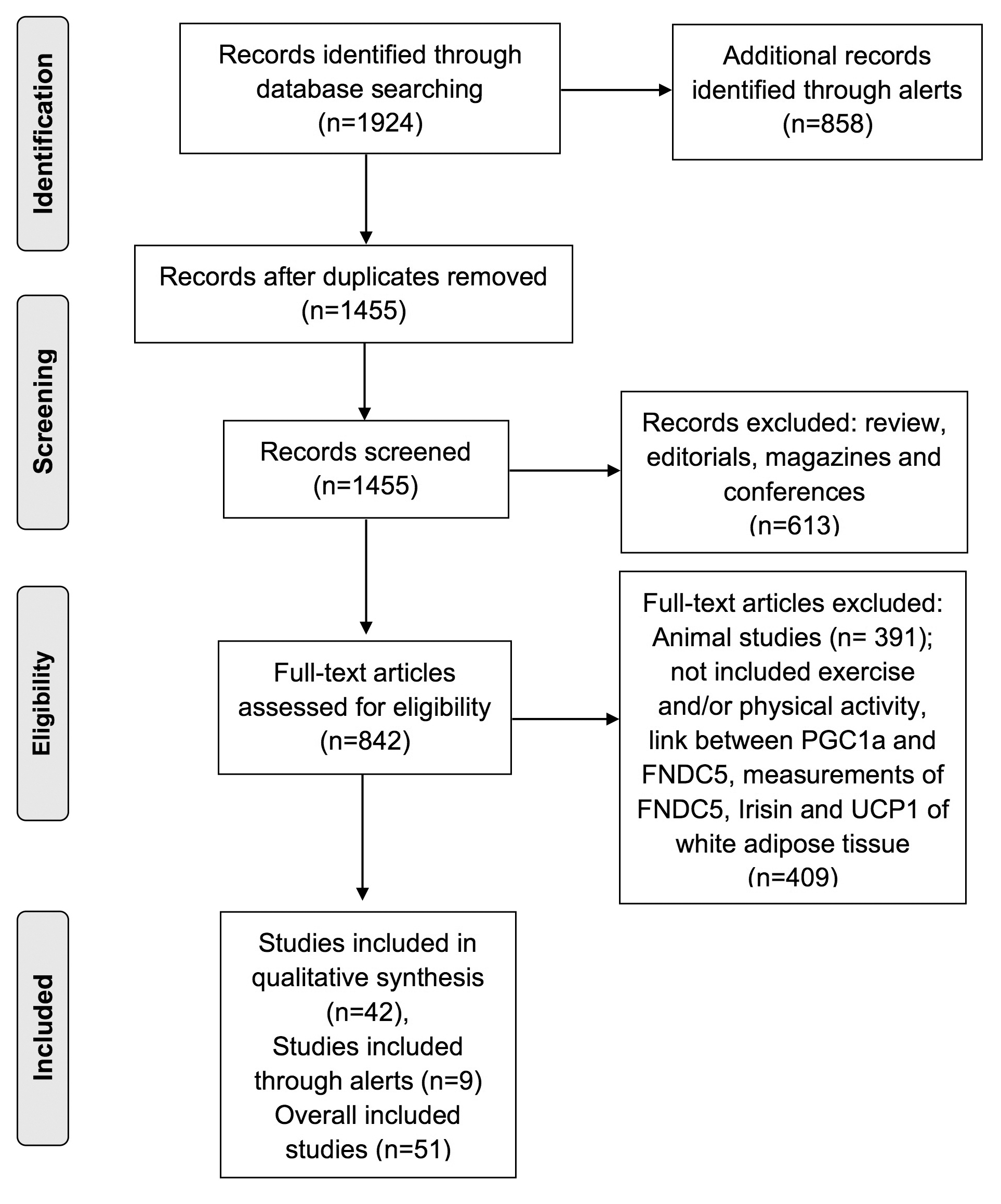

Supplement: Supplementary file 4 [file f1000research-6-12649-s0003.tgz › 4933dafc-e166-4d5e-a5e0-1000610e843f.tif]

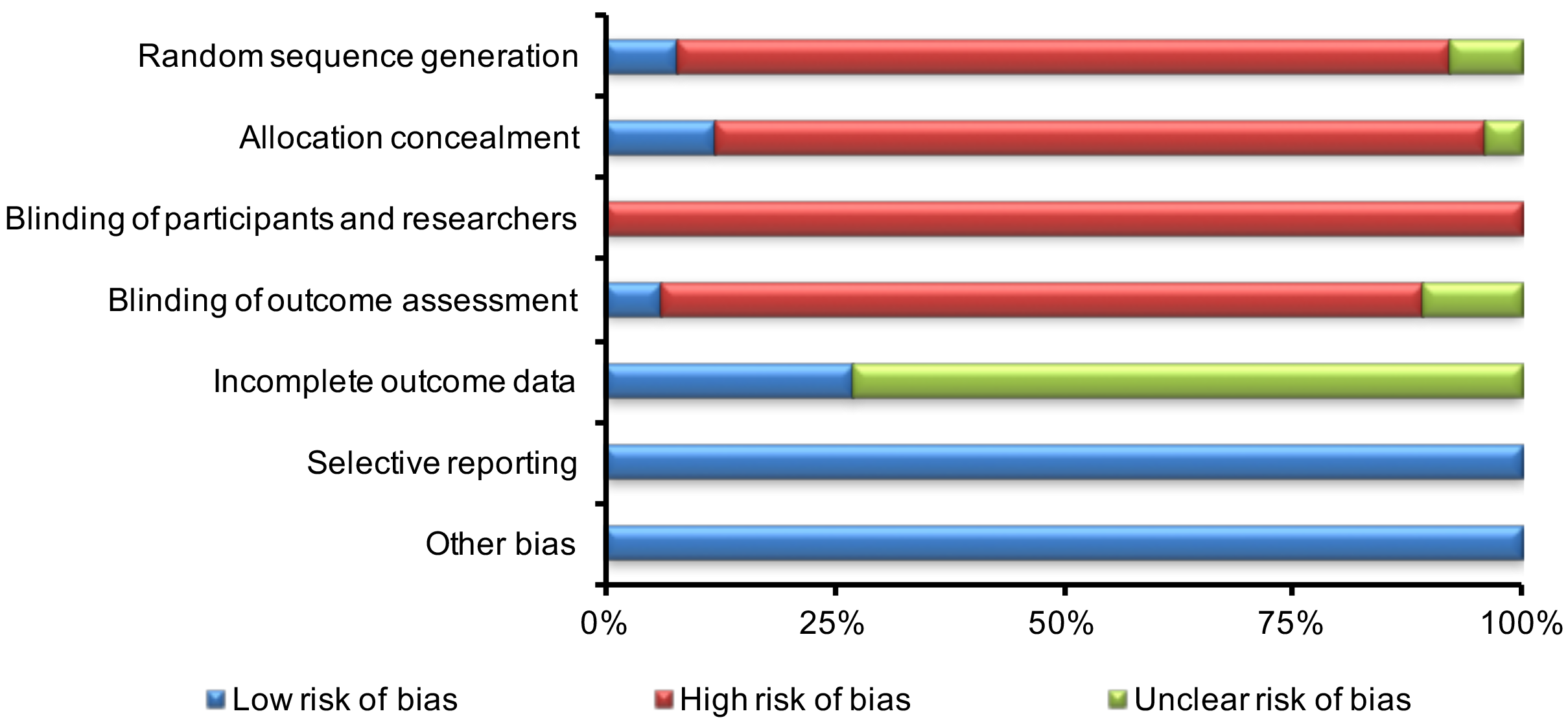

Supplement: Supplementary file 5 [file f1000research-6-12649-s0004.tgz › 6fe30747-0602-49ba-824a-bc43a4c01ac4.tif]
