# Supplementary material for: Effects of physical activity on the link between PGC-1a and FNDC5 in muscle, circulating Ιrisin and UCP1 of white adipocytes in humans: A systematic review
Source: F1000Res. 2017 May 26;6:286. Originally published 2017 Mar 17. [Version 2] doi: 10.12688/f1000research.11107.2 (PMC5461915; doi:10.12688/f1000research.11107.2)
Supplement: Peer review history

# Peer review: Referee response for version 2

**DOI:** 10.5256/f1000research.12649.r23048

**Published:** 2017-05-31

**Authors:** Elke Albrecht, Steffen Maak

**Affiliations:** 1 Institute of Muscle Biology and Growth, Leibniz Institute for Farm Animal Biology, Dummerstorf, Germany; 2 Institute of Muscle Biology and Growth, Leibniz Institute for Farm Animal Biology, Dummerstorf, Germany

The authors have addressed all of our concerns in an adequate manner. Importantly, all uncertainties resulting from methodological problems of the irisin measurements in the included studies are now clearly named in the re-structured discussion.

 There are three minor issues left:

 (I) + (II) The authors used the term “molecular weight” two times (page 16, last paragraph) when addressing “concentrations” or “circulating levels” of irisin.

 (III) Again, not the similar molecular weight of irisin compared to leptin, insulin, and resistin makes a biological function of irisin likely but the similar circulating concentrations (discussion page 15).

We have read this submission. We believe that we have an appropriate level of expertise to confirm that it is of an acceptable scientific standard.

# Peer review: Referee response for version 1

**DOI:** 10.5256/f1000research.11982.r21091

**Published:** 2017-03-29

**Authors:** Fabian Sanchis-Gomar

**Affiliations:** 1 Leon H. Charney Division of Cardiology, New York University School of Medicine, New York, NY, USA; 2 Department of Physiology, Faculty of Medicine, University of Valencia and Fundación Investigación Hospital Clínico Universitario/INCLIVA, Valencia, Spain

In this manuscript, the authors analyzed the effects of physical activity on the connection between muscle peroxisome proliferator-activated receptor gamma coactivator 1-alpha (PGC-1α) and fibronectin type III domain-containing protein 5 (FNDC5), circulating Irisin and uncoupling protein one (UCP1) of white adipocytes in humans through a systematic review. The authors review the evidence connecting PGC-1α, FNDC5, Irisin and UCP1 with physical exercise, which might play an important role in diabetes and obesity. Moreover, they have addressed a controversial topic since, though Irisin was initially described as a hormone potentially regulated by exercise and also with a potential role in obesity and diabetes, conflicting results have been reported. There is an ongoing intense debate on different results from different Irisin assays. In effect, in 2014, we were the first to underline those discrepancies and inconsistencies. For that reason, I feel this review is timed appropriately and is interesting for the scientific community. The review is effectively organized and the sequence of points is logical, and follows PRISMA guideline.

 In my opinion, both results and discussion are properly presented, although the discussion section should be slightly shortened. Also, references 8 and 87 are not needed. I recommend changing PGC-1a by PGC-1α throughout the manuscript. Furthermore, the following manuscript that was recently published deserves to be included and discussed: Perakakis et al. (2017) ^1^.

I have read this submission. I believe that I have an appropriate level of expertise to confirm that it is of an acceptable scientific standard.

## References

[1] PerakakisNTriantafyllouGAFernández-RealJMHuhJYParkKHSeufertJMantzorosCS: Physiology and role of irisin in glucose homeostasis. *Nat Rev Endocrinol*.2017; 10.1038/nrendo.2016.221 10.1038/nrendo.2016.221 28211512PMC5878942

# Response

**Published:** 2017-05-22

**Authors:** PETROS DINAS

**Affiliations:** FAME LABORATORY, UNIVERSITY OF THESSALY, Greece

We thank you very much for your encouraging comments.

As per your suggestion references 8 and 87 have been removed. We have also changed PGC-1a with PGC-1α throughout the manuscript. Finally, the reference you suggested has been included in the discussion section along with a relevant comment (Page 37). ****

# Peer review: Referee response for version 1

**DOI:** 10.5256/f1000research.11982.r21095

**Published:** 2017-03-27

**Authors:** Elke Albrecht, Steffen Maak

**Affiliations:** 1 Institute of Muscle Biology and Growth, Leibniz Institute for Farm Animal Biology, Dummerstorf, Germany; 2 Institute of Muscle Biology and Growth, Leibniz Institute for Farm Animal Biology, Dummerstorf, Germany

The presented review is timely and of common interest since energy expenditure by “browning” of white adipocytes may be a mechanism to reduce body weight and a potential way to fight obesity in humans. Beneficial effects of exercise on metabolism are long known although not completely understood. A recently proposed pathway linked physical activity with increased PGC1-alpha and FNDC5. Cleavage of the transmembrane factor FNDC5 results in circulating irisin which in turn starts a sequence to induce a brown-like phenotype in white adipocytes. Consequently, numerous studies focused on this proposed link in different experimental settings in humans. The presented review systematically retrieved and analyzed such studies to further elucidate different steps of the proposed cascade PGC1-alpha – FNDC5 – Irisin – UCP1.

 The value of this review bases on its rigorous quality assessment of the included studies. The authors analyzed all published reports with established tools on the risk of bias and the quality of the data. This part of the analysis demonstrates a rather low quality of many studies hampering the deduction of valid conclusions. The analysis of the reported results is given in different sections considering the analyzed parameters and the design of the studies. Results on PGC1-alpha and FNDC5 as well as on UCP1 are presented and discussed in an appropriate manner.

 In contrast, the data on irisin are much more problematic. As the authors mention in the discussion, the missing validation of the used ELISA kits for irisin is a major issue in these studies. The values vary greatly and, more importantly, are mostly higher by magnitudes than the values determined by mass spectrometry. In some studies irisin was measured with different assays: Kurdiova et al. (ref. 18) measured irisin with a RIA (RK-067-16, Phoenix) and a subset of their samples additionally with ELISA (EK-067-29, Phoenix) and concluded “… The correlation between both assays was very weak, and definitely not admissible for two products that claimed to measure the concentrations of the same molecule…”. Albrecht et al. (ref. 27) re-analyzed all samples from Norheim et al. (ref. 17, EK-067-52) with the ELISA of Adipogen and found no correlation between both measurements (r = 0.03). Finally, Montes-Nieto et al. (2016) ^1^ analyzed irisin in human plasma with two different lots of EK-067-29 (Phoenix) and stated an almost complete lack of agreement between the data. Latter reference should be included in this review. These results cast doubts at least on all irisin levels determined with those kits used in more than half of the included studies. Consequently, a meaningful discussion of the data is hardly possible.

 The authors should mention these points at a prominent position and shorten the detailed discussion of the – most likely – invalid ELISA/RIA results from the included studies.

 Moreover, it should be noted that the studies using mass spectrometry delivered contradictory results. Boström et al. (ref. 2) and Lee et al. (ref. 25) used an antibody (Abcam, now discontinued) recognizing a peptide of FNDC5 which is not part of the secreted irisin to identify the bands subjected to mass spectrometry whereas Jedrychowski et al. (ref. 74) employed an antibody of Adipogen against the irisin peptide. This explains the discrepancy in the molecular weight of the analyzed peptides (~ 22 kDa [ref. 2, 25] vs. ~ 12 kDa [ref. 74]).

 There is some redundancy in the description of results and repetitions in the discussion which should be omitted. It is e.g. not necessary to repeat several times the values for irisin determined with mass spectrometry. It would increase readability if more summarized results are presented.

 Additionally, some minor points should be corrected:

 Introduction

- Reference 8 is not suited to support this statement because it comments the results of Boström et al. and is no independent confirmation

Table 1, page 5

- Aydin 2013: They used EK-067-52 for measurement of serum irisin. The given product H-067-17 is an antibody for immuno-histochemistry

page 6

- Moienneia 2016: The correct name of the company is CUSABIO and it is based in China – the name and affiliation is wrong in the original publication.
- Khodadadi 2014: They mention CUSABIO in the article. This company provides only one ELISA for irisin therefore it is likely that they used the same test like Moienneia 2016.

page 8

- Moienneia 2016: See above.

page 10

- Kwasniewska 2016: Typo in the name. They used the irisin ELISA produced by the Czech company BioVendor. The given Scottish company does not sell irisin ELISAs. This is misleadingly described in the original article.

Discussion, page 16

- Reference 87 is not suited to support the statements regarding mass spectrometry (3 times). I guess ref. 87 (Lee DV et al.) was mixed up with ref. 25 (Lee P et al.). Please check whether ref. 87 is needed at all.

page 17

- Reference 26 is not suited to support the statement regarding mass spectrometry. It was probably mixed up with ref. 25.

 

 Taken together, this systematic review is a valuable contribution to guide through the confusing literature concerning the relationship between exercise and the proposed PGC1-alpha – FNDC5 – irisin – axis. The evaluation of the included studies with well-acknowledged quality measures adds additional value and makes the article unique in the reviewed field.

We have read this submission. We believe that we have an appropriate level of expertise to confirm that it is of an acceptable scientific standard, however we have significant reservations, as outlined above.

## References

[1] Montes-NietoRMartínez-GarcíaMÁLuque-RamírezMEscobar-MorrealeHF: Differences in analytical and biological results between older and newer lots of a widely used irisin immunoassay question the validity of previous studies. *Clin Chem Lab Med*.2016;54(7) : 10.1515/cclm-2015-1071 e199-201 10.1515/cclm-2015-1071 26677893

# Response

**Published:** 2017-05-22

**Authors:** PETROS DINAS

**Affiliations:** FAME LABORATORY, UNIVERSITY OF THESSALY, Greece

We have implemented the suggested information regarding Irisin identification in content with the already existing argument in the text. Also, we removed the detailed discussion of the most likely invalid ELISA (Page 37).  We would like to mention that we completely agree with the Reviewer that a meaningful discussion regarding the effects of physical activity on Irisin is hardly possible, given the problematic methods used for Irisin identification. In this regard, we conclude that we cannot form any firm conclusion. Furthermore, we highlight the fact (Conclusions section) that even though we used a well-established methodology for systematic reviews, we had to additionally consider the validity and accuracy of the methods used in the included studies to avoid misleading conclusions. Finally, we have mentioned all this information that was suggested by the reviewer including the reference by Montes-Nieto et al. (2016) (Page 35).

We have implemented the information (Page 36) that the studies using mass spectrometry delivered contradictory results.

The results section is formed based on the mechanism that this systematic review examined (i.e. PGC-1a and FNDC5 in muscle, Irisin and UCP1 in white adipocytes) to directly reflect to the aim of the study. In addition, we report the results considering the different kind of exercises (i.e. acute and chronic exercise), while we separately present the relationships of the examined factors with physical activity levels. We believe that this is particularly important to increase clarity in the presented outcomes in line with the aim of our systematic review. However, considering your comment, we removed the repetition from the results section (i.e. values for Irisin determined with mass spectrometry) (Pages 31-32).  We also removed repetition from the discussion section as per your suggestion (Pages 34).

We removed the reference 8 from the relevant statement.

We have corrected the Table 1 (Pages 9, 10, 11, 15 and 18)

Thank you for bringing to our attention that reference 87 was mixed up with reference 25. We have now corrected this in the text.

We have removed the reference 26 that was mixed up with reference 25.

.
